# Impact of telemedicine on metabolic control and hospitalization of peritoneal dialysis patients during the COVID-19 pandemic: a national multicentric cohort study

**DOI:** 10.1590/2175-8239-JBN-2021-0113

**Published:** 2022-02-23

**Authors:** Nicole Iasmin Magario Tabuti, Caio Pellizzari, Henrique Carrascossi, Viviane Calice-Silva, Ana Figueiredo, Gina Moreno Gordon, Gilson Biangini, Mario Ernesto Rodrigues, Dayana Bitencourt Dias, Dirceu Reis da Silva, Thyago Proença de Moraes

**Affiliations:** 1 Santa Casa de Misericórdia de Curitiba, PR, Brasil.; 2 Instituto do Rim Carrascossi, Araraquara, SP, Brasil.; 3 Fundação Pró-rim, Joinville, SC, Brasil.; 4 Universidade da Região de Joinville, Joinville, SC, Brasil; 5 Pontifícia Universidade Católica do Rio Grande do Sul, Porto Alegre, RS, Brasil.; 6 Fundação Pró-Renal, Curitiba, PR, Brasil.; 7 Instituto do Rim de Curitiba, Curitiba, PR, Brasil.; 8 Renal Care, Brasilia, DF, Brasil.; 9 Universidade Estadual de São Paulo, Botucatu, SP, Brasil.; 10 Instituto de Doenças Renais, Porto Alegre, RS, Brasil.; 11 Pontifícia Universidade Católica do Paraná, Curitiba, PR, Brasil.

**Keywords:** Kidney Failure, Chronic, Biomarkers, Peritonitis, Mortality, Falência Renal Crônica, Biomarcadores, Peritonite, Mortalidade

## Abstract

**Introduction:**

The coronavirus-19 pandemic threatens the lives of all people, but results in higher mortality rates for patients with end-stage kidney disease (ESKD) including those on peritoneal dialysis (PD). Telemedicine was the main alternative to reduce exposure to the virus, but it was introduced in the Brazil without proper training.

**Objective:**

To investigate the impact of telemedicine on metabolic control, peritonitis rates, and hospitalization in PD patients during the pandemic.

**Methods:**

This was a retrospective multicenter cohort study. We included all adult patients on chronic PD from 9 clinics selected by convenience during the pandemic. The outcomes of interest were measured and compared between before and after switching to telemedicine using repeated measure analysis and multilevel Poisson regression.

**Results:**

The study included 747 patients with a mean age of 59.7±16.6 years, of whom 53.7% were male and 40.8% had diabetes. Biochemical parameters including hemoglobin, potassium, phosphate, calcium, and urea serum levels did not change significantly after transition to telemedicine. There was no association between telemedicine and peritonitis rates. In contrast, hospitalization rates increased significantly in the telemedicine period. The incidence rate ratio (IRR) for hospitalization in the telemedicine period was 1.54 (95%CI 1.10-2.17; p 0.012) and 1.57 (95%CI 1.12-2.21; p 0.009) in the mixed-effects Poisson regression before and after adjustment for the presence of confounders. Admissions for hypervolemia and infections not related to PD doubled after transition to telemedicine.

**Conclusion:**

The implementation of telemedicine without proper training may lead to an increase in adverse events in PD patients.

## Resumo

**Introdução:**

A pandemia do coronavírus-19 ameaça a vida de todas as pessoas, mas resulta em uma alta taxa de mortalidade em pacientes com doença renal em estágio terminal (DRET), incluindo aqueles em diálise peritoneal (DP). A telemedicina foi a principal alternativa para reduzir a exposição ao vírus, mas foi introduzida no Brasil sem treinamento adequado.

**Objetivo:**

Investigar o impacto da telemedicina no controle metabólico, taxas de peritonite e hospitalização em pacientes em DP na pandemia.

**Métodos:**

Estudo de coorte multicêntrico retrospectivo. Incluímos todos os pacientes adultos em DP crônica de 9 clínicas selecionadas por conveniência durante a pandemia. Desfechos de interesse foram medidos e comparados entre antes e depois da mudança para telemedicina usando análise de medidas repetidas e regressão multinível de Poisson.

**Resultados:**

Incluiu-se 747 pacientes com idade média de 59,7±16,6 anos, sendo 53,7% homens e 40,8% diabéticos. Parâmetros bioquímicos, incluindo níveis séricos de hemoglobina, potássio, fosfato, cálcio e ureia não mudaram significativamente após transição para telemedicina. Não houve associação entre telemedicina e taxas de peritonite. Em contraste, taxas de hospitalização aumentaram significativamente no período de telemedicina. A razão de taxas de incidência (RTI) para internação no período de telemedicina foi 1,54 (IC95% 1,10-2,17; p 0,012) e 1,57 (IC95% 1,12-2,21; p 0,009) na regressão multinível de Poisson antes e após ajuste para presença de fatores de confusão. As internações por hipervolemia e infecções não relacionadas à DP dobraram após transição para telemedicina.

**Conclusão:**

A implementação da telemedicina sem treinamento adequado pode levar ao aumento de eventos adversos em pacientes em DP.

## Introduction

The COVID-19 pandemic caused drastic changes in the world. The disease caused by a virus with extremely high contagiousness can have high lethality depending on the predisposing medical conditions of the patient^1^. Individuals with chronic kidney disease (CKD), particularly those with end-stage kidney disease (ESKD), are at a higher risk of developing severe respiratory complications, being hospitalization, staying long periods of time in the intensive care unit, and even death^2,3^. In this context, peritoneal dialysis (PD) has attracted more attention during this time because, as a home treatment, it reduces patient exposure to the virus. However, PD patients may also be exposed to the virus, and the most critical time for these patients is the routine monthly visit to the clinic. Therefore, the implementation of telehealth has been proposed.

Worldwide, medical teams felt compelled to adopt pragmatic measures to minimize the exposure of PD patients to COVID-19 and started to use telemedicine to minimize this risk^4-6^. However, in Brazil, the use of telemedicine was restricted by law before COVID-19 and there was no experience with this new approach to patient care. In this context, longer periods of time without an in-person visit and lack of training in telemedicine may delay the identification of clinical problems that could otherwise be immediately diagnosed timely with an in-person visit. Furthermore, skipping visits may give PD patients the false impression that their conditions are less severe than they actually are, and problems related to diet and drug adherence may arise.

Despite this important and potentially life-saving initiative to reduce the risk of a COVID-19 infection, to our knowledge, no study evaluated the impact of telemedicine on outcomes in PD patients. The aim of our study was to analyze the short-term incidence of metabolic disorders and hospitalization rates with the use of telemedicine during the pandemic.

## Methods

This was a multicentric, retrospective cohort of 9 Brazilian PD centers. Data were retrieved from the records of patients on chronic PD between January 2020 and June 2020. For scientific purposes, the study was divided into 2 phases: the first phase corresponded to the 2-month period before the clinic switched to telemedicine and phase 2 was the 2 months after transition to telemedicine. Both phases are divided by a 1-month period during which the transition occurred. The “transition” month was characterized by some patients being seen in-person at clinics, while others had already started telemedicine, and a group that simply missed the visit in that month for fear of the pandemic.

Demographic data were collected at baseline (first month of phase 1). Clinical and laboratory data were collected along the study until the end of follow-up. The biomarkers selected for the study were hemoglobin, potassium, phosphate, calcium, and urea. These exams were chosen because they are part of the monthly routine exams required by local regulatory policies. These biomarkers were treated as continuous variables but also categorized as follow: (a) for hemoglobin: percentage of patients below 10 g/dL; (b) for phosphate: percentage of patients with P >5.5 mg/dL; (c) for potassium: percentage of patient below 3.5 mEq/L and above 5.5 mEq/L.

Data were also collected on center characteristics, which included center size (as measured by the total number of patients on chronic PD), penetrance of PD (percentage of patients on PD and HD), the percentage of patients with previous hemodialysis, the presence of a nurse dedicated only to PD (yes or no), the nurses-to-patients ratio, the percentage of patients on APD, the type of contact with the patient during the telemedicine period (phone or teleconference with video), and the support provided by the multidisciplinary team during the pandemic (yes or no). The topics evaluated in each center in each online visit is shown in supplementary Table S1.

### Information collected during the online contact

Each center adapted to telemedicine as best as it could. Given the urgency of avoiding patient exposure to COVID-19, there was no time to standardize procedures. The routine interviews were performed monthly, and both nephrologists and nurses interviewed patients in all centers except one, in which only nephrologists interviewed patients. Questions to patients were related to: (a) adherence to medication and PD prescription (e.g. are you doing your dialysis and taking your medications as prescribed?; Did you skip any day of dialysis during the last month?); (b) ultrafiltration rate (patients were asked to provide data on daily UF in milliliters); (c) diuresis volume in 24 h in a random day of the month; (d) presence of edema (yes or no); (e) presence of dyspnea/shortness of breath (yes or no); (f) presence of purulent discharge at the exit site (yes/no); (g) glycemic control for diabetic patients (episodes of hypoglycemia/ elevated glucose levels?); (h) blood pressure control (episodes of hypotension/ uncontrolled hypertension?); (i) water/liquids intake (are you adhering to water and salt restriction?); (j) dietetic management; (k) weight gain/loss; (l) abdominal pain (yes or no; if yes, clinical characteristics of the pain and dialysate appearance; and (m) presence of any other symptom not covered in previous questions (Do you have any other symptom interfering in your daily activities?).

This study was performed in accordance with the Declaration of Helsinki and approved by the local Ethical Committee (approval n° 4.089.635).

### Statistics

Continuous variables were expressed as mean ± SD or median and interquartile range, whereas categorical variables were expressed as frequencies and percentages. For comparison of continuous variables between before and after telemedicine, the paired T test was used, and when variables were categorized into binary variables, the chi-square test was used. For hospitalization, as an event that can occur more than once, a Poisson model was used.

A mixed-effect Poisson regression (patient in the first level and clinic in the second level) analysis was used to identify factors associated to clinical practice. For the multivariable model, we selected variables with an alpha level below 0.10 in the univariate analysis (Table S1). We then performed a backward elimination removing one at a time if it did not contribute to the regression equation. Statistical significance was set at p<0.05. All analyses were performed with STATA14.

## Results

### General characteristics

The study included 747 patients from 9 PD dialysis clinics. The mean age of study participants was 59.7±16.6 years, 53.7% was male, and 40.8% had diabetes. The demographic data of our study population are similar to those of the country’s population on PD^7^. Table 1 shows the general demographic and clinical characteristics of our population. The size of the participating clinics ranged from 18 to 256 patients, the penetrance of PD varied from 17 to 78%, 95% had nurses engaged full-time with PD, and regarding telemedicine, only 2 clinics used videoconference with their patients, whereas the remaining 7 clinics conducted the regular follow-ups by telephone. During the course of the study, 8 patients (1%) were diagnosed with COVID, 50% of whom (n=4) died as a result of the virus.

**Table 1:** General characteristics of the population

Variable	Frequency or median (IQR)
**Patient**	
Age > 65 years	44%
Caretaker (yes)	55%
Diabetes (yes)	41%
Gender (male)	54%
Literacy (years)	8 (4-11)
Missed one consult (yes)	10%
Modality (APD)	97%
Previous HD (yes)	35%
Race (White)	65%
Vintage (years)	20 (9-35)
**Center**	
Center size (n)	140 (91-221)
Multidisciplinary (yes)	42%
Exclusive nurse	95%
PD penetrance (each 10 patients)	27 (25-30)
Use of videoconference (yes)	6%

Legend: IQR-Interquartile range

### Metabolic parameters

**Figure 1:**
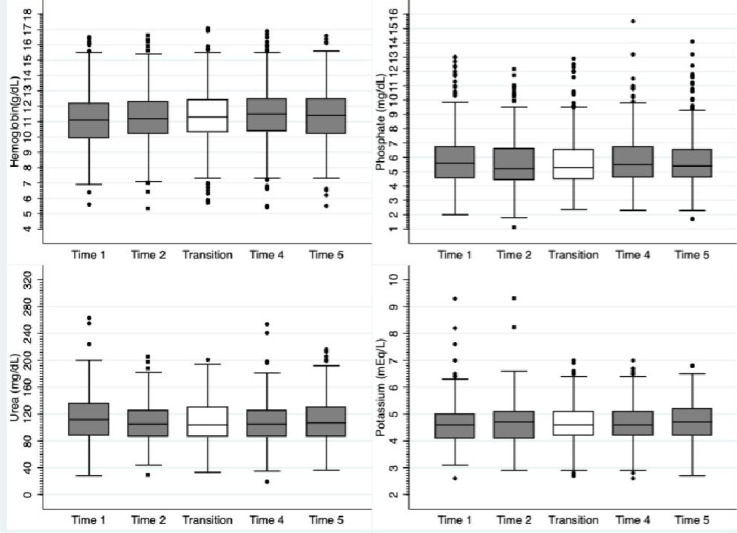
Variation on mean values of metabolic parameters after transition to telemedicine.

**Figure 2:**
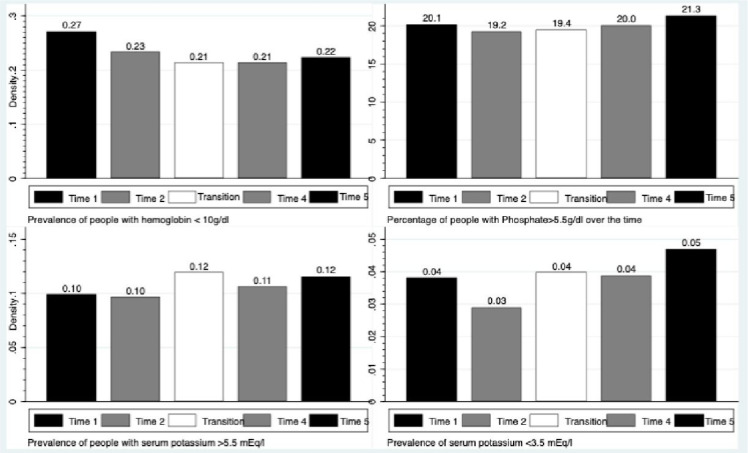
Prevalence of abnormal metabolic parameters after transition to telemedicine.

The proportion of patients with hemoglobin levels below 10 g/dL was 27% at baseline and decreased to 19% by the end of the follow-up. Mean hemoglobin levels were 11.0±1.8 g/dL at baseline and 11.2±1.9 at the end of the study (Figures 1 and 2).

**Figure 3:**
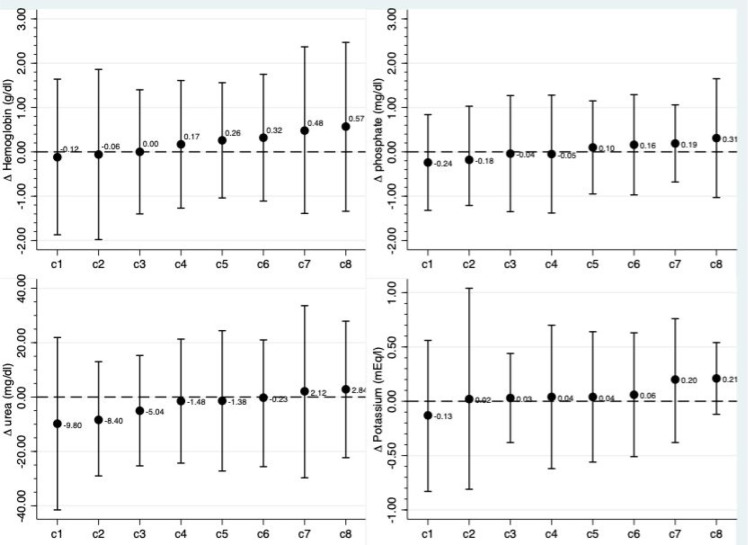
Variation on mean values of metabolic parameters after transition to telemedicine by centers.

Legend: mean difference (post - pre) per center after transition to telemedicine. Values were organized in ascending order.

In contrast, the variations of potassium and phosphate levels were very small (Figure 3). Mean potassium was 4.6±0.8 at baseline and 4.6±0.7 by the end of the study, whereas the mean value for phosphate was 5.9±1.9 g/dL at the beginning and remained stable at 5.9±1.8 g/dL during the study. In terms of metabolic disturbances, hyperkalemia was identified in 10% at baseline and 12% at the end of follow-up, while hypokalemia was 4 and 5%, respectively. For phosphate, 20% had values above 5 mg/dL at baseline and 21% at the end of the study. Finally, we evaluated serum urea levels, and a small change from 116 to 112 mg/dl was found.

### Hospitalization rates

There were 55 admissions in 50 patients (5 patients were admitted twice) during the first phase of the study, while in the second phase, the numbers increased to 91 admissions in 82 patients (9 patients were admitted twice). The overall hospitalization rate was 17.8 (15.0 - 21.1) admission per 1000 patient-months. In both phases of the study, admissions were considered in all possible hospitals, not only in the hospitals with PD programs.

The risk of hospitalization increased after the transition to telemedicine before and after exclusion of the hospitalizations related to COVID-19. Unadjusted IRR was 1.65 (1.18-2.31) considering the admissions by COVID-19 and 1.54 (1.10-2.16) after excluding the hospitalizations by COVID-19. Regarding causes of hospitalization (Table 2), the largest increase occurred due to cases associated to hypervolemia and infections not related to PD (i.e., peritonitis). The absolute number of cases for both causes doubled after the transition to telemedicine. Details on the causes of hospitalizations are shown in the supplementary material (Table S2). Of the 16 cases of infections not related to PD, only 3 were caused by bacterial pneumonia.

**Table 2:** Causes of hospitalization

Causes	Phase 1 % (n)	Phase 2 % (n)
Cardiovascular	20 (11)	16 (15)
Catheter dysfunction	5 (3)	3 (3)
COVID	-	7 (6)
Hypervolemia	9 (5)	12 (11)
Infection unrelated to PD	9 (5)	12 (11)
Metabolic disorders	11 (6)	8 (7)
Peritonitis	18 (10)	14 (13)
Others	22 (12)	21 (19)
Missing	5 (3)	7 (6)

**Figure 4:**
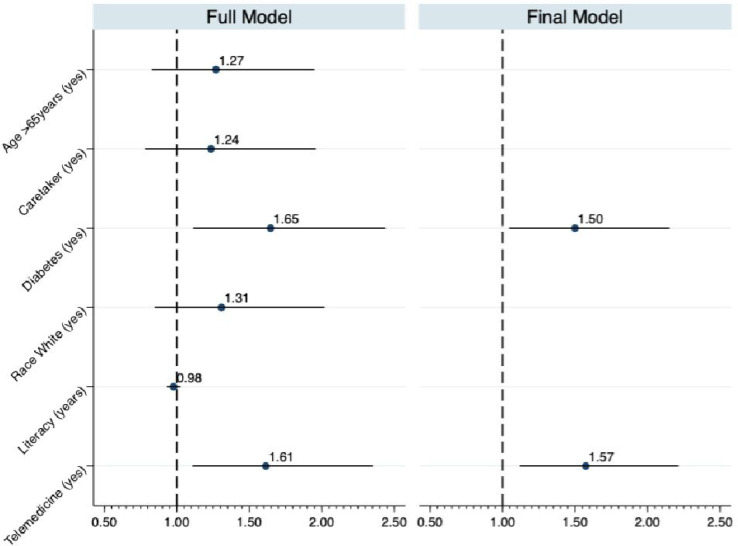
Multivariable mixed-effect Poisson regression: risk factors for hospitalization.

We identified a series of factors associated to an increased risk of hospitalization in the univariate analysis (Table S2), which included: patients with a caretaker (IRR 1.54; 95% CI 1.07-2.22); diabetes (IRR 1.49; 95% CI 1.05-2.12), and White race (IRR 1.48; 95% CI 1.00-2.18) (Figure 4). No variable related to the center was associated with hospitalization. They were included in the initial multivariate analysis (full model), and after backward elimination, the model ended with presence of a caretaker as the only covariate. The IRR for the telemedicine period was 1.54 (95% CI 1.10-2.17) and the IRR for presence of a caretaker was 1.57(95% CI 1.07-2.28).

### Peritonitis and exit-site infection rates

We found that 33 (4%) of the 747 patients were diagnosed with peritonitis in the first phase of the study and 37 (5%) had peritonitis during the 2 months after the implementation of remote monitoring. There was no significant difference between the rate of peritonitis in both periods (p=0.60). We also found that 2% of PD patients were diagnosed with exist-site infection in the 2 months prior to the transition to telemedicine, and this number remained stable at 2% during the 2 months following the transition. The difference between the two periods was also not significant (p=0.69).

## Discussion

To our knowledge, this is the first study to analyze the impact of telemedicine on outcomes of PD patients. In summary, a significant increase in hospitalization rates occurred with the use of telemedicine during the COVID-19 pandemic in a large multicentric cohort of chronic PD patients.

In the early 2020, the world was struck by the COVID-19 pandemic. By October, according to the WHO, more than 72 million people had been infected and diagnosed and more than 1.6 million people had died. In Brazil, over 5 million cases had been detected by the end of the same period causing the death of around 155,000 people. The majority of cases are patients with mild or asymptomatic disease, but severe and life-threatening presentations are not uncommon in absolute numbers because of the high transmissibility and consequent high number of people contaminated with the virus^1^. In particular, the presence of some conditions and comorbidities predisposes the patients to a severe presentation^1-3^.

Patients with CKD are at a high risk of developing severe COVID-19 as demonstrated in a meta-analysis of 73 studies (OR=1.84; 95% CI 1.47-2.30)^8^. Not only is the risk of a severe presentation higher, but also COVID-19-related death is higher in patients with CKD and especially in individuals with ESKD^9,10^. The hazard ratio for mortality was 3.69 (95% CI 3.09-4.39) in a large cohort of almost 24,000 patients on dialysis^9^. In a recent description of outcomes on hemodialysis patients from Brazil, the fatality rate associated to COVID-19 was almost 28%^10^. In PD, data are scarcer. In a cohort of 848 patients from Wuhan, the incidence of symptomatic COVID-19 was similar to that of the general population, but the fatality rate was 25% (2 out of 8 patients)^11^. In our study, 8 of the 747 patients included were infected with COVID-19 and the fatality rate was 50% (4 out of 8 patients).

In hopes of containing the risk of COVID-19, several measures were taken worldwide, either based on previous experiences with other deadly viruses or simply based on common sense in the absence of evidence. Measures such as environment disinfection, use of personal protective equipment, screening of potentially infected patients, and efforts to isolate them have been widely used^12^. For PD patients, several centers also opted for telemedicine use to further reduce exposure to the virus^4-6^.

Telemedicine, defined as the exchange of medical information between different locations, allows patients and the medical team to be in different rooms and even cities. Ideally, telemedicine should be performed using a videoconference, which requires a good and stable internet connection, a computer with a good performance, and a specific software to exchange information in real time. However, in developing countries, such equipment is not available to most people. An alternative is to contact patients by phone and rely only on patient information without actually seeing them. This has been the alternative for the majority of patients on PD in Brazil.

Our study found that monitoring and controlling basic biochemical parameters important for all dialysis patients is relatively simple to obtain. No change of clinical significance was observed before and after changing to telemedicine. When we designed the study for the management of anemia and potassium levels, minimal changes were expected. For anemia, the main influence in the short term is the prescription and adjustment of erythropoiesis-stimulating agents, which can be easily done remotely. The same is true for control of potassium serum levels, which are not significantly altered by blockade of the renin-angiotensin-aldosterone system and changes in the patient’s diet, provided dialysis adherence remains constant^13,14^. However, given the educed nutritional support during the pandemic, we expect an increase in phosphate serum levels, but again the values remained stable.

In contrast to the task of remotely monitoring biochemical parameters and PD technique, monitoring other clinical parameters can provide another level of complexity and can be difficult to identify when the patient is not literally in front of the medical team. Dialysis patients in particular are prone to hypervolemia, which imposes significant challenges to diagnosis in normal clinical practice. This is certainly intensified when we evaluate patients remotely without using the proper tools. This was one of our hypotheses, and it was confirmed by a two-fold increase in the number of hospitalizations. Except for metabolic alterations, other conditions also increased during remote monitoring, including infections unrelated to PD, peritonitis, and cardiovascular causes. It is important to note that the absolute number of PD-related infections was small (before and after telemedicine) and any difference (or lack of difference in this case) should be interpreted with caution.

It is noteworthy that diabetes was the only other factor associated with increased risk of hospitalization during the pandemic. A survey with 1,562 Brazilian patients with diabetes found that measures taken by states to protect this high-risk group left most of them unprotected. The authors reported that these measures contributed to an increase in glycemic levels and/or its variability. In our study, we did not collect data on glycemic control, and we can only speculate that similar problems may have contributed to the higher risk of hospitalization in our diabetic patients.

Finally, our study indicates that despite the enthusiasm for new technologies and the incorporation of telemedicine into our practice (an irreversible movement), changes should always be planned carefully, taking into account all variables and adapting to different realities. Nevertheless, it is important to point out other factors that may have influenced our results and that are not related to the way we used telemedicine during the study. The Brazilian public health system (known as SUS) provides selected medicines for the treatment of CKD and other comorbidities as hypertension and diabetes, free of charge. However, it was not recorded whether patients avoided going to the primary healthcare system to take their medication for fear of possible infection with COVID. This possibility is partially mitigated by the fact that clinics asked about adherence to all drugs. Nevertheless, our study has some limitations including all those related to any retrospective observational study and the small number of patients treated by videoconference. We did not have data on social dislocation or details of treatment with other specialties. In addition, we were unable to obtain data on changes in ultrafiltration rate and/or in daily urine output. In contrast, the large number of PD patients included and the robust statistics that took into account center characteristics are strengths of our work.

In conclusion, the number of hospitalizations unrelated to COVID increased during the pandemic, and the implementation of telemedicine without proper training and equipment, although necessary in the current scenario, may be harmful to PD patients.

## Supplementary Material

The following online material is available for this article:

Table S1 - Topics discussed during the online visit by each center.

Table S2 - Univariate results for the risk ratio of hospitalization: mixed-effect Poisson regression.
