# Supplementary material for: Impact of telemedicine on metabolic control and hospitalization of peritoneal dialysis patients during the COVID-19 pandemic: a national multicentric cohort study
Source: J Bras Nefrol. 2022 Feb 23;44(4):473–81. doi: 10.1590/2175-8239-JBN-2021-0113 (PMC9838680; doi:10.1590/2175-8239-JBN-2021-0113)
Supplement: Supplementary file 1 [file 2175-8239-jbn-2021-0113-suppl1.pdf]

Braz. J. Nephrol. (J. Bras. Nefrol.)
https://doi.org/10.1590/2175-8239-JBN-2021-0113

Supplementary Material to the "Impact of telemedicine on metabolic control and hospitalization of peritoneal dialysis patients during the COVID-19 pandemic: a national multicentric cohort study"

Table S1 - Topics discussed during the online visit by each center.

**Topic**	**C1**	**C2**	**C3**	**C4**	**C5**	**C6**	**C7**	**C8**	**C9**
Adherence to PD prescription	✓	✓	✓	✓	✓	✓	✓	✓	✓
Ultrafiltration rate	✓	✓	✓	✓	✓	✓	✓	✓	✓
Diuresis (quantification)	✓	❋	✓	✓	✓	❋	✓	✓	✓
Edema (presence or not)	✓	✓	✓	✓	✓	✓	✓	✓	✓
Dyspnea (presence or not)	✓	✓	✓	✓	✓	✓	✓	✓	✓
Exit-site purulent drainage (presence or not)	✓	✓	✓	✓	✓	✓	✓	✓	✓
List of medications	✓	✓	✓	✓	✓	✓	✓	✓	✓
Glycemic control (if diabetes)	✓	✓	✓	✓	✓	✓	✓	✓	✓
Blood pressure control (if hypertension)	✓	✓	✓	✓	✓	✓	✓	✓	✓
Water/liquid intake	✓	✓	❋	✓	✓	✓	✓	✓	✓
Dietetic control	✓	❋	✓	❋	❋	❋	✓	✓	✓
Weight	✓	✓	❋	❋	❋	❋	✓	✓	✓
Abdominal pain (presence or not)	✓	✓	✓	✓	✓	✓	✓	✓	✓
Subjective well-being or aditional issues	✓	✓	✓	✓	✓	✓	✓	✓	✓
Use of videoconference	❋	❋	❋	❋	❋	❋	✓	❋	❋
Profile of the interviewer									
- Nephrologist	✓	✓	✓	✓	✓	✓	✓	✓	✓
- PD Nurse	✓	❋	✓	✓	✓	✓	✓	✓	✓

Legend: PD-peritoneal dialysis.
