# Supplementary material for: Impact of telemedicine on metabolic control and hospitalization of peritoneal dialysis patients during the COVID-19 pandemic: a national multicentric cohort study
Source: J Bras Nefrol. 2022 Feb 23;44(4):473–81. doi: 10.1590/2175-8239-JBN-2021-0113 (PMC9838680; doi:10.1590/2175-8239-JBN-2021-0113)
Supplement: Supplementary file 2 [file 2175-8239-jbn-2021-0113-suppl2.pdf]

Braz. J. Nephrol. (J. Bras. Nefrol.)
https://doi.org/10.1590/2175-8239-JBN-2021-0113

Supplementary Material to the "Impact of telemedicine on metabolic control and hospitalization of peritoneal dialysis patients during the COVID-19 pandemic: a national multicentric cohort study"

Table S2 - Univariate results for the incidence risk ratio of hospitalization: mixed-effect Poisson regression.

**Variable**	**IRR**	**CI 95%**	**p**
Patient level			
Age > 65 years	**1.42**	**0.99-2.03**	**0.05**
Caretaker (yes)	**1.54**	**1.07-2.22**	**0.02**
Diabetes (yes)	**1.49**	**1.05-2.12**	**0.03**
Gender (male)	**1.00**	**0.70-1.43**	**0.97**
Literacy (years)	**0.96**	**0.92-1.01**	**0.10**
Miss one consult (yes)	**0.94**	**0.51-1.72**	**0.84**
Modality (APD)	**0.75**	**0.30-1.89**	**0.55**
Previous HD (yes)	**1.11**	**0.76-1.63**	**0.58**
Race (White)	**1.48**	**1.00-2.18**	**0.05**
Vintage (years)	**1.05**	**0.96-1.15**	**0.28**
Center level			
Center size (n)	**1.00**	**0.99-1.01**	**0.37**
Multidisciplinary (yes)	**1.40**	**0.77-2.56**	**0.27**
Nurse (exclusive)	**0.76**	**0.25-2.29**	**0.62**
PD Penetrance (each 10 patients)	**0.99**	**0.86-1.14**	**0.93**
Use of videoconference (yes)	**1.03**	**0.37-2.89**	**0.95**
